# Biotic aspects of suspended solid reduction in sedimentation ponds

**DOI:** 10.1007/s11356-024-35475-0

**Published:** 2024-11-21

**Authors:** Andrzej Skrzypczak, Renata Tandyrak

**Affiliations:** Institute of Engineering and Environmental Protection, Faculty of Geoengineering, University of Warmia and Mazury, Oczapowskiego St. 5, 10-719 Olsztyn, Poland

**Keywords:** Artificial ecosystems, Fish habitat, Mine waters, Technological sedimentation, Water treatment

## Abstract

The reduction of suspended solids is an important aspect of water management, especially in the mining industry. The ecological potential of sedimentation ponds has been identified, but many aspects of the coexistence of ecological and technological functions of these reservoirs are still unresolved. The aim of the study was to determine the effectiveness of suspended solid reduction in an open pit mine drainage system under the influence of biotic factors, including fish, taking into account the hydrochemical background. The dynamics of biotic factors and the sedimentation process were identified using the normalized total suspended solid reduction index (TE) and the trophic group status (TGS) indices of planktivorous, benthivorous, and carnivorous fish. The hydrochemical background in both ponds provided similar habitat conditions for biota and suspended solid reduction. Although a moderately trophic ecosystem has developed, the typical relationship between water transparency (SDD) and chlorophyll a concentration characteristic of natural waters was not observed. The sedimentation complex was found to be highly effective in reducing total suspended solids by 89–93%. Spatial variation of sedimentation rates was related to the ichthyobiotic factor, mainly to the biomass of bentophages and the status of this trophic group in the fish community. Stable relationships in the structure of the ichthyofauna with a balanced proportion of planktivorous fishes favored the sedimentation process. The results obtained are part of the development of research on the possibility of combining the technological function of artificial ponds with other applications and ecological benefits for industrialized areas.

**Supplementary information:**

The online version contains supplementary material available at 10.1007/s11356-024-35475-0.

## Introduction

Sedimentation ponds are an important part of the dewatering system that is critical to the operation of an open pit mine. Therefore, one of the most important aspects of open pit mining is the problem of water management. The drainage system collects groundwater and water from precipitation. Before it is discharged to the external natural hydrological system and surface water, it usually needs to be pre-treated to remove excess suspended solids. This is because the chemical quality of the suspended solids can affect water quality in areas surrounding the mine (Lottermoser 2007; Butler and Ford 2017; Agboola et al. 2020).

There is considerable evidence that high concentrations of suspended sediment, and its deposition can disrupt many plant and animal life processes (Henley et al. 2000). Suspended sediment causes a range of environmental damage, including smothering of benthic organisms, irritation of fish gills, and transport of sorbed contaminants. Much of the impact while sediment remains suspended is related to its light attenuation, which reduces visibility in the water and light availability for photosynthesis (Davies-Colley and Smith 2001; Wood and Smith 2022). In addition, the problem of acidification of mine waters and higher than normal trace element contamination is quite widespread (Geller et al. 2013). Therefore, hydrochemical analysis should always be conducted in parallel with other ecological studies, as heavy metal mobility is determined by many environmental factors (Wang and Tan 2019). However, such risks are more characteristic of mine waters from deep dewatering, while they usually do not occur in surface dewatering (Napiórkowska-Krzebietke et al. 2023). Napiórkowska-Krzebietke and Skrzypczak (2021) have shown that the sediment ponds of a surface drainage system can also have a good ecological potential. It has been scientifically proven that under certain conditions the sediment pond complex can provide a safe environment for fish and aquatic invertebrates, including extensive aquaculture (Skrzypczak and Napiórkowska-Krzebietke 2020). Fish farming in mine waters for food and recreation is technically feasible (Mallo et al. 2010; McCullough et al. 2020) and acceptable in terms of food quality and safety standards (D'Souza et al. 2004). It follows that sedimentation ponds may share a technological function with an ecological function. However, aspects of the coexistence of these functions remain an unresolved issue. This is due to the peculiarities of sedimentation processes.

The operation of a clarifier is based on the slow sedimentation of substances and their accumulation at the bottom, where they are partly decomposed and partly covered and immobilized by mineral particles. The rate of sedimentation depends on the properties of the suspended particles, i.e., their size, volumetric weight, and shape, as well as a number of variable characteristics of the water environment, such as the density and viscosity of the liquid and turbulence. The rate of deposition of suspended particles increases with increasing water temperature, while turbulent motions retard sedimentation. The load deposited on the bottom is a potential source of secondary water pollution. Re-pollution can be very rapid and can occur as a result of increased water flow or increased turbulence (Garcia 2008; Ostrovsky et al. 2014). Thus, the sedimentation process can be perturbed by a number of factors of different origin. Therefore, an identified research gap is to what extent do ecological function and biotic factors, in particular fish, influence the technological effectiveness of sedimentation ponds? The problem is important because open sedimentation ponds are influenced by natural ecological processes and are spontaneously colonized by numerous living organisms.

The aim of the study was to determine the influence of the ichthyotic factor on the rate and efficiency of reduction of suspended solids in the sedimentation pond complex, taking into account the abiotic and biotic background. The rationale behind the objective undertaken is to understand the processes of effective combination of the technological function with other uses in terms of sustainable water management, including as an ecological benefit. In the case of industrialized areas and areas without natural water reservoirs, it is particularly important in social and natural aspects.

## Materials and methods

### Study area

**Fig. 1:**
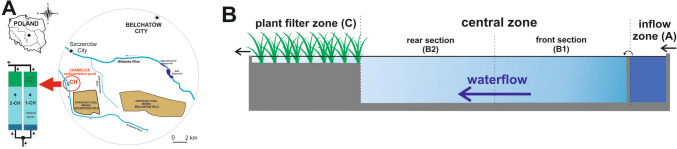
Location of the study area (**A**) and longitudinal section diagram of the Chabielice sedimentation pond (ponds 1-CH and 2-CH) of the Bełchatów open-cast mine (**B**)

As a case study, sedimentation ponds supplied with water from the dewatering system of “Bełchatów,” the largest open pit lignite mine in Poland, which has been in operation since 1975, were selected. The sedimentation complex named Chabielice is located at the WGS84 point: 51°15′58.9″ N, 19°06′24.3″ E. It consists of two separate earthen ponds (1-CH and 2-CH) that are parallel to each other and separated by dikes ≈ 3.5 m high (Fig. 1). The total area of these complex sedimentation ponds is 16.02 ha, and the area of each pond is 8.01 ha. The surface drainage system removes water from precipitation and the pit surface. This water is pumped to the surface through pumping stations and then conveyed to the sedimentation pond complex through an open channel. The separation well determines that a similar volume of water with identical physicochemical parameters supplies the ponds. With a maximum allowable inflow of 1.8 m^3^ s^−1^, the water retention time in each pond is about 16 h. The complex of two ponds is used for water treatment by gravitational sedimentation of inorganic and organic suspended solids in water. Each pond consists of three functional zones. The first inflow zone (A) with an area of about 0.15 ha is used to remove the coarsest fractions of suspended solids. Then, the water flows through an overflow comb baffle and enters the central zone, which is the main technological zone of the pond with a maximum depth of 2.4 m. It is functionally divided into a front section (B1) and a rear section (B2). The process of sedimentation and water clarification is completed in the plant filter zone (C). This filter (area 0.80 ha; average depth 0.50 m) contains macrophytes such as *Phragmites australis* (Cav.) Trin. ex Steud., *Typha latifolia* L., *Glyceria maxima* (Hartm.) Holmb., *Phalaris arundinacea* L., *Acorus calamus* L., and *Carex acutiformis* L. (Skrzypczak and Napiórkowska-Krzebietke 2020). Behind the filter, there is a drain and the water flows into a collective drain.

### Sampling and analysis of physicochemical parameters

Field surveys were conducted in three annual cycles, i.e., 2018, 2019, and 2020, including four sampling regimes in each year: spring (April), summer (June and August), and fall (October). In order to study the linear distribution of total suspended solid concentration and structure, water samples were taken from 1-m depth in the central part of the inflow zone (A), the front part (B1) and the back part (B2) of the central zone, and in the central part of the plant filter zone (C). Water samples for other physicochemical parameters were taken in the middle of the central zone of each pond (1-CH and 2-CH) of the Chabielice sedimentation complex as representative sampling points, at a depth of 1 m below the water surface. A total of 160 water samples were collected for laboratory testing and analysis during the study period.

Water temperature (T) and dissolved oxygen (DO) were measured with the YSI 6600 V2 Multi-Parameter Water Quality Sonde, while pH and total dissolved solids (TDS) were measured with the HQ30D Digital Multimeter. In each chamber, water transparency was measured with a Secchi disk (SDD). The selection of ions and trace elements for the determinations was made on the basis of the results of the hydrogeochemical background performed for the area of the mining activities (Pękala 2014). Such a range of hydroanalyses corresponds to the cyclic monitoring of mine water quality supervised by the Polish National Hydrogeological Service (Gogacz 2004). Laboratory analyses included: total suspended solids (TSS), inorganic suspended solids (ISS), organic suspended solids (OSS)—gravimetric analysis (PN-EN 872:2007), turbidity (MERCK SQ118), calcium (Ca), and bicarbonate (HCO_3_-)–titrimetric analysis. Total phosphorus (TP) was determined colorimetrically with a NANOCOLOR spectrophotometer (Macherey–Nagel GmbH&Co. KG, Düren, Germany) after mineralisation with H_2_SO_4_ and K_2_S_2_O_8_, with the use of ammonium molybdate and SnCl_2_ (*λ* = 690 nm). Total nitrogen (TN) and nonvolatile total organic carbon (TOC) were determined in an IL 550 TOC-TN analyzer (HACH Inc., Loveland, CO, USA). Chlorophyll *a* (Chl *a*) concentration was determined after filtration through a Whatman GF/C glass fiber filter and extraction with acetone. Hydrochemical analyses were performed according to APHA guidelines (APHA 2000).

### Biosampling

Zooplankton samples were collected four times a year, including the spring (April), summer (June and August), and fall (October) seasons of each year of the study. Samples were collected using the Ruttner sampler (5 L) from the representative site in the middle of the central zone of each pond at 1-m depth (1-CH and 2-CH). The zooplankton sample material of 10 L was sieved through a plankton net (mesh size of 25 μm) for both quantitative and qualitative analyses. Lugol’s standard solution (I2 in KI) was used to preserve the samples. Identification and measurements of zooplankton were performed using the Zeiss AXIO Imager microscope and available references (Błędzki and Rybak 2016; Ejsmont-Karabin et al. 2004).

Zooplankton density was determined using a Sedgewick rafter. The density (ind. L^−1^) and total biomass (mg L^−1^) of zooplankton were calculated using different methods. The biomass of planktonic crustaceans was determined according to Bottrell et al. (1976) and Ruttner-Kolisko (1977), while the individual body weights of rotifers were based on the standard wet weights according to Ejsmont-Karabin (1998).

In each of the ponds, fish samples were taken by gill net twice a year, i.e., in spring (April) and in fall (October), according to the standard protocol (CEN 2005) and standard sampling (Deceliere-Vergẻs et al. 2009). Referring to the concept of functional roles of species in ecosystems (Franco et al. 2008), the study of the dynamics of the fish community was carried out by dividing it into trophic groups, including planktivorous, benthivorous, and carnivorous fish. This division is a simplification of complex food webs and identifies how fish use ecosystem resources regardless of taxonomic identity (Elliott et al. 2007; Brejão et al. 2013). The planktivorous fish group is mainly active in the water tone and therefore in the water zone directly associated with sedimentation. The benthivorous fishes are active in the bottom sediments and therefore in the zone where sediment particles are deposited. Carnivorous fishes are responsible for controlling the abundance of planktivorous and benthivorous fishes. The group of planktivorous fish included both species that feed exclusively on plankton, i.e., species for which zooplankton is the main food during their entire life cycle (e.g., sunbleak and *Leucaspius delineatus*), as well as juveniles of all species in the first and second year of life, which have the greatest impact on the plankton fauna (Biró and Vörös 1990). Studies to date clearly indicate that such fish (individual body weight < 10 g) are caught by the smallest mesh size of gillnets (Olin et al. 2015). Therefore, all fish caught by panels with mesh sizes of 5.0, 6.25, 8.0, and 10.0 mm were included in the planktivorous fishes (the effective catch panels are 15 m^−2^ of each gillnet). Fish caught by panels with larger meshes, i.e., 12.5, 15.5, 19.5, 24.0, 29.0, 35.0, 43.0 and 55.0 mm (the effective catch panels is 30 m^−2^ of each gillnet), were classified into two separate trophic groups, i.e., invertebrate-eating (benthivorous) and fish-eating (carnivorous). The exception was perch—a facultative piscivore in which invertebrates are also an important part of the diet (Svanbäck and Eklöv 2004). Therefore, perch specimens in the individual weight range of 10–200 g (caught with panels with mesh sizes of 12.5, 15.5, 19.5, and 24.0 mm—the effective catch panels are 15 m^−2^ of each gillnet) were classified as benthivorous fish, and those above 200 g as carnivorous fish (caught with panels with mesh sizes of 29.0, 35.0, 43.0, and 55.0 mm—the effective catch panels are 15 m^−2^ of each gillnet) (Kurkilahti et al. 2015). Ichthyobiotic indices were calculated according to the CEN standard, including density (number per unit effort, NPUE) and biomass (weight per unit effort, WPUE) of fish (Olin et al. 2015). Density (NPUE, ind. m^−2^) and biomass (WPUE, g m^−2^) of the different trophic groups of fish were calculated in relation to the area of the effective panels of the gillnets. The final NPUE and WPUE indices for each trophic group were standardized by relating them to a statistical area of 10 m^−2^ of gillnets.

### Analytical indicators

In order to achieve the intended research goals, it was deemed necessary to define standardized indicators that would objectively reflect the dynamics of sedimentation processes and biotic factors. To determine the linear sedimentation rate of total suspended solids, the efficiency index of total suspended solids concentration, expressed as trap efficiency (TE), was determined. TE is the proportion of incoming sediment that is deposited or trapped in a pond (Tan et al. 2019; Verstraeten and Poesen 2000):

TE = (TSS_inflow_-TSS_outflow_)/TSS_inflow_.

where: TSS_inflow_ is the concentration of total suspended solids flowing into the sedimentation pond chamber and TSS_outflow_ is the concentration of total suspended solids leaving the sedimentation pond chamber with the outgoing water;

TE values range from 0.0 to 1.0. A higher value of the index indicates a greater efficiency in reducing the total suspended solids (1.0 = 100%).

In order to identify changes in the fish communities and to analyze the relationships between the three trophic groups, the special index of trophic group status (TGS) was determined. It was calculated for each fish trophic group based on the biomass index (WPUE) according to the formula:

Plankti-TGS = (∑Planktivorous WPUE – (∑Benthivorous WPUE + ∑Carnivorous WPUE)) / ∑Total WPUE;

Benthi-TGS = (∑Benthivorous WPUE – (∑Planktivorous WPUE + ∑Carnivorous WPUE)) / ∑Total WPUE;

Carni-TGS = (∑Carnivorous WPUE – (∑Planktivorous WPUE + ∑Benthivorous WPUE)) / ∑Total WPUE;

TGS values range from − 1.0 to 1.0. A decreasing TGS index from 0 to − 1.0 indicates a decrease in the trophic group status of fish in the ichthyofauna community. An increasing TGS index from 0 to 1.0 indicates increasing dominance of a particular trophic group over other groups.

### Statistical procedures

Abiotic data, including suspended sediment concentration and other physicochemical parameters of the water, and biotic data, including qualitative-quantitative indicators of the zooplankton and fish community, were tested for normality using the Shapiro–Wilk test, which confirmed that the data were not normally distributed. Therefore, the non-parametric analysis of variance (ANOVA) with the Kruskal–Wallis test (to compare more than two independent samples) was the primary tool used to determine the statistical significance of differences between environmental variables between the annual study periods in the two chambers of the tailings pond. The significance level was set at *p* < 0.05. These analyses were performed using Statistica software (ver. 13.3 for Windows, Statsoft, Tulsa).

The dissimilarity between the two ponds during the growing seasons of 2018, 2019, and 2020 was tested with the non-metric multidimensional scaling (NMDS) ordination analysis based on biotic and abiotic data, including the prepared indicators of total suspended solid reduction efficiency and indicators of the status of trophic groups of ichthyofauna. NMDS analysis has been effectively used to explain complex trophic relationships in aquatic ecosystems (Le Gall et al. 2024). The Bray–Curtis distance measure, two axes and stress formula type 2 were used for log-transformed abiotic and biotic parameters (ter Braak and Šmilauer 2018). The analysis was carried out using Canoco 5.

## Results and discussion

### Sedimentation rate of suspended solids and hydrochemical background of this process

Most of the monitored physicochemical indicators in the Chabielice sedimentation complex were characterized by moderate and non-significant variability (Table S1). However, the Kruskal–Wallis test confirmed the higher turbidity of water in both ponds in 2018, i.e., 1-CH- 92.6 NTU; 2-CH- 128.6 NTU (*H* = 17.52; *p* = 0.0141), compared to definitely lower and not statistically different the turbidity indices in 2019–2020. The variability of the water transparency index in the last year of the study was also shown. In 2020, the Secchi disk visibility (SDD) in 1-CH reached 0.95 m and was significantly higher than in 2-CH 0.70 m (*H* = 16.23; *p* = 0.0272). Other indicators can be considered stable despite the variability of the recorded concentrations. Water temperature and dissolved oxygen concentration reflect seasonal variations in the annual cycle (May–October). In subsequent years of the study, water temperature in both sedimentation ponds was similar, with average values between ponds differing slightly in the range of 0.1–0.3 °C. The water was well oxygenated, and differences in dissolved oxygen concentrations between the ponds were not statistically significant. During 2018–2020, the average TDS concentration ranged from 523.8 ± 102.9 mg L^−1^ (pond 2-CH in 2020) to 584.2 ± 109.7 mg L^−1^ (pond 1-CH in 2018). The hard-water character of the sedimentation complex environment is indicated by the mean concentrations of bicarbonate ions, i.e., from 237.1 ± 21.8 mg L^−1^ (pond 1-CH in 2020) to 291.1 ± 31.2 mg L^−1^ (pond 2-CH in 2018), and calcium ions, i.e., from 98.3 ± 22.6 mg L^−1^ (1-CH 2019) to 117.7 ± 21.6 mg L^−1^ (2-CH in 2020). The mean concentration of total phosphorus had a variation ranging from 0.052 ± 0.006 mg L^−1^ (2-CH in 2019) to 0.089 ± 0.010 mg L^−1^ (1-CH in 2018). Correlation coefficient between turbidity index and total suspended solid concentration was *R*^2^ = 0.8549 in pond 1-CH and indicated a satisfactory adjustment of these values, however, this relationship was not demonstrated in 2-CH (*R*^2^ = 0.3105). Similarly, the relationship between TP concentration and turbidity in 1-CH (*R*^2^ = 0.5759) was demonstrated, but not confirmed in pond 2-CH (0.2623). No correlation between SDD results and chlorophyll *a* concentration was found in both ponds. The greatest variation in mean values versus standard deviation was found in chlorophyll *a* concentration. This indicator ranged from 3.75 ± 3.37 µg L^−1^ (pond 1-CH in 2019) to 4.99 ± 2.53 µg L^−1^ (pond 2-CH in 2018).

The course of sedimentation processes is reflected in the indicators of the total concentration of total suspended solids (TSS) concentration and its structure (ISS-OSS ratio) in the longitudinal section of each of the pond chambers. In the alluvial zone (A) and in the front part of the central zone (B1) no statistical differentiation of the suspended solids indices was found (Table 1). In zone A, the mean concentration of suspended particles ranged from 67.3 ± 7.2 mg L^−1^ (pond 1-CH in 2019) to 72.4 ± 7.8 mg L^−1^ (pond 2-CH in 2019). In zone B1 of the 1-CH, the TSS concentration ranged from 46.9 ± 6.2 mg L^−1^ (in 2019) to 51.7 ± 6.7 mg L^−1^ (in 2018), and in the 2-CH from 45.3 ± 5.9 mg L^−1^ (in 2020) to 54.5 ± 6.5 mg L^−1^ (in 2019). In the last part of the central zone (B2) and in the plant filter zone (C), the average concentration of total suspended solids in pond 2-CH was, respectively, 34.1 ± 6.8 mg L^−1^ and 7.7 ± 1.8 mg L^−1^, respectively, and were higher than in pond 1-CH, i.e.: 22.0 ± 3.2 mg L^−1^ and 4.8 ± 0.4 mg L^−1^, respectively (Fig. S1). In zones A and B1, the inorganic fraction dominated in the suspension, while in zones B2 and C, the organic fraction increased. In pond 1-CH, the average percentage of the inorganic fraction ranged from 70.5% in the inflow zone to 8.9% in the plant filter zone. In 2-CH, these values were respectively 69.1% and 10.9%, respectively. Statistical differentiation of the structure of the suspension was observed only in the central zone of each pond, i.e., in the front (B1) and end (B2) parts. In zone B1 of 1-CH in 2018 and 2020, the average percentage of inorganic fraction was 51.4 ± 4.7% and 50.3 ± 3.5%, and at the same time, it was statistically lower than in pond 2-CH in each year of observation. Throughout the study period in zone B2, the percentage of organic suspension was significantly higher in 1-CH than in 2-CH (Table 1).

**Table 1:** Concentration and structure of suspended solids (*TSS* total, *ISS* inorganic, and *OSS* organic) in the Chabielice sedimentation ponds (A—inflow zone; B1—front of central zone; B2—back of central zone; and C—plant filter zone) in the years 2018–2020 (mean of twelve samples, ± SD)

		Pond 1-CH			Pond 2-CH		
Zone	Parameters	2018	2019	2020	2018	2019	2020
A	TSS (mg L^−1^)	70.4 ± 8.4	67.3 ± 7.2	69.6 ± 7.9	68.5 ± 8.1	72.4 ± 7.8	71.9 ± 8.2
	ISS (%)	69.4 ± 3.2	73.5 ± 3.1	68.7 ± 3.4	67.9 ± 3.3	72.9 ± 3.7	66.4 ± 3.9
	OSS (%)	30.6 ± 3.2	26.5 ± 3.1	31.3 ± 3.4	32.1 ± 3.3	27.1 ± 3.7	33.6 ± 3.9
B1	TSS (mg L^−1^)	51.7 ± 6.7	46.9 ± 6.2	48.2 ± 6.9	45.3 ± 5.9	54.5 ± 6.5	51.2 ± 6.3
	^**1**^ISS (%)	51.4^**A**^ ± 4.7	57.9^**AB**^ ± 4.9	50.3^**A**^ ± 3.5	62.1^**B**^ ± 4.8	63.1^**B**^ ± 4.7	61.5^**B**^ ± 3.9
	^**2**^OSS (%)	48.6^**A**^ ± 4.7	42.1^**AB**^ ± 4.9	49.7^**A**^ ± 3.5	37.9^**B**^ ± 4.8	36.9^**B**^ ± 4.7	38.5^**B**^ ± 3.9
B2	^**3**^TSS (mg L^−1^)	24.1^**A**^ ± 3.4	19.7^**A**^ ± 3.1	22.3^**A**^ ± 3.2	28.7^**AB**^ ± 5.9	35.2^**B**^ ± 6.8	38.4^**B**^ ± 7.7
	^**4**^ISS (%)	28.7^**A**^ ± 3.3	25.4^**A**^ ± 3.2	27.6^**A**^ ± 3.5	45.7^**B**^ ± 5.9	43.9^**B**^ ± 5.3	46.3^**B**^ ± 5.8
	^**5**^OSS (%)	71.3^**A**^ ± 3.3	74.6^**A**^ ± 3.2	72.4^**A**^ ± 3.5	54.3^**B**^ ± 5.9	56.1^**B**^ ± 5.3	53.7^**B**^ ± 5.8
C	^**6**^TSS (mg L^−1^)	4.4^**A**^ ± 0.3	4.9^**A**^ ± 0.4	5.1^**A**^ ± 0.5	5.8^**AB**^ ± 1.2	8.3^**B**^ ± 1.9	8.9^**B**^ ± 2.4
	ISS (%)	10.3 ± 2.4	8.7 ± 2.2	7.8 ± 2.1	12.8 ± 2.6	9.3 ± 2.3	10.6 ± 2.5
	OSS (%)	89.7 ± 2.4	91.3 ± 2.2	92.2 ± 2.1	87.2 ± 2.6	90.7 ± 2.3	89.4 ± 2.5

Values in the rows with the different superscripts are significantly different among the years by non-parametric Kruskal–Wallis test (ANOVA, *N* = 30, df = 5, *p* < 0.05); ^**1**^*H* = 17.53, *p* = 0.0103; ^**2**^*H* = 17.59, *p* = 0.0101; ^**3**^*H* = 16.25, *p* = 0.0221; ^**4**^*H* = 18.42, *p* = 0.0093; ^**5**^*H* = 18.74, *p* = 0.0086; ^**6**^*H* = 16.49, *p* = 0.0209

The trapping efficiency coefficient (TE) of suspended solids in the longitudinal section of the Chabielice sedimentation pond ranged from 0.878 ± 0.018 in 2-CH in 2020 to 0.938 ± 0.004 in 1-CH in 2018 (Table 2). In the absence of statistical variation throughout the observation period, it was characterized by stability. Such stability of the TE coefficient was also observed between the inflow zone (A) and the front part of the central zone (B1). Its magnitude varied from 0.247 ± 0.035 (chamber 2-CH in 2019) to 0.324 ± 0.169 (2-CH in 2018). However, in the central zone, i.e., between the front and rear sections, the suspended matter trapping coefficient was statistically higher in 1-CH throughout the observation period (*H* = 21.04; *p* = 0.0049). The TE in 1-CH ranged from 0.536 ± 0.011 (year 2020) to 0.569 ± 0.123 (year 2019), and in 2-CH the ratio obtained values ranging from 0.237 ± 0.224 in 2020 to 0.365 ± 0.098 in 2018. The highest rate of reduction of suspended solids was observed between the back of the central zone (B2) and the plant filter zone (C). TE values ranged from 0.751 ± 0.022 (1-CH in 2019) to 0.816 ± 0.035 (1-CH in 2018).

**Table 2:** Unit values of the trapping efficiency (TE) coefficient of sediment suspensions in the longitudinal section of the Chabielice sedimentation complex (ponds 1-CH and CH-2; A—inflow zone; B1—front of the central zone: B2—back of central zone; C—plant filter zone) in the years 2018–2020 (mean of twelve samples, ± SD)

Zone	Pond 1-CH			Pond 2-CH		
	2018	2019	2020	2018	2019	2020
A/B1	0.259 ± 0.131	0.301 ± 0.077	0.296 ± 0.166	0.324 ± 0.169	0.247 ± 0.035	0.166 ± 0.015
B1/B2*	0.537^A^ ± 0.016	0.569^A^ ± 0.123	0.536^A^ ± 0.011	0.365^B^ ± 0.098	0.358^B^ ± 0.059	0.237^C^ ± 0.224
B2/C	0.816 ± 0.035	0.751 ± 0.022	0.769 ± 0.047	0.798 ± 0.021	0.764 ± 0.019	0.754 ± 0.099
Total A/C	0.938 ± 0.004	0.927 ± 0.011	0.928 ± 0.001	0.913 ± 0.027	0.886 ± 0.014	0.878 ± 0.018

*Values in the rows with the different superscripts are significantly different among the years by non-parametric Kruskal–Wallis test (ANOVA, *N* = 30, df = 5, *H* = 21.04, *p* = 0.0049)

Regardless of the TE indices, the two chambers of the Chabielice sedimentation pond differed significantly in the concentration of suspended solids in the rear part of the central zone and in the plant filter zone according to the 1-CH < 2-CH rule. In addition, the structure of suspended solids in the central zone was different, i.e., for ISS: 1-CH < 2-CH; for OSS: 1-CH > 2-CH. Available research (Asthana and Khare 2022; Verstraeten and Poesen 2000) indicates that coarser mineral material sediments faster, while very fine material requires a long residence time. Therefore, the TE rate depends on the particle size and structure of the incoming sediment and the residence time. The studied ponds of the sedimentation complex were supplied with water of the same physical and chemical parameters. In addition, the retention time in both ponds of the sedimentation complex did not differ and was about 16 h. According to Maggi (2009), the sedimentation velocity of suspended solids is strongly correlated with the proportion of the mineral fraction of suspended solids, while it is not significantly correlated with the total volume of suspended solids (and their size), suggesting a revision of current methods for assessing suspended solids deposition based solely on particle size. Studies by Garcia (2008) and Ostrovsky et al. (2014) indicate that suspended solids concentrations in coal mine tailings ponds are affected by even small changes in salinity. Increases in total dissolved solids (TDS) concentration and electrical conductivity (EC) decrease the repulsion coefficient and increase the sedimentation rate. Another important factor is the temperature of the water due to the increasing density difference between water and suspended particles. However, these factors did not differ between the ponds. Assuming that all the physical factors influencing the sedimentation effect (height of the water layer, properties of the sedimentation particles, flow turbulence, wind influence, temperature and density of the water) were the same in both sedimentation ponds, the reasons for the lower efficiency of sedimentation should be sought in the hydrobiotic conditions.

### Biotic factors and how they interact to reduce suspended solids

Zooplankton showed very high seasonal variability each year with Rotifer dominance, especially in pond 2-CH where it ranged from 94.1 ± 0.9 to 95.7 ± 1.4% (Table S2). The average density of planktonic organisms in pond 2-CH was several to tens of times higher than in pond 1-CH, and simultaneously decreased from 14,154 ± 27,008 mg L^−1^ in 2018 to 4259 ± 7051 mg L^−1^ in 2020 (Table 3). There was also a decrease in mean zooplankton biomass during this period, ranging from 3.22 to 0.98 mg L^−1^. In 1-CH, there was a lower dynamics of zooplankton variability, both in terms of biomass (from 0.39 to 0.43 mg L^−1^) and density (from 743 to 883 ind. L^−1^).

**Table 3:** Biological indices of zooplankton density and biomass and fish (NPUE, number per unit effort; WPUE, weight per unit effort) in the Chabielice sedimentation complex (1-CH and 2-CH) in 2018–2020 (mean of eight samples, ± SD)

Parameters		1-CH			2-CH		
		2018	2019	2020	2018	2019	2020
Zooplankton	Density (ind. L^−1^)	883 ± 1139	865 ± 1002	743 ± 711	14,154 ± 27,008	5986 ± 10,589	4259 ± 7051
	Biomass (mg L^−1)^	0.41 ± 0.35	0.43 ± 0.42	0.39 ± 0.33	3.22 ± 4.08	1.22 ± 2.19	0.98 ± 1.13
Planktivorous fish*	^**1**^ NPUE (ind. 10 m^−2^)	89.2^A^ ± 37.5	92.6^A^ ± 39.7	51.3^B^ ± 21.7	64.8^AB^ ± 25.3	73.7^AB^ ± 32.1	87.8^A^ ± 36.3
	^**2**^ WPUE (g 10 m^−2^)	513.7^A^ ± 104.2	408.4^A^ ± 112.9	275.4^B^ ± 76.3	302.2^B^ ± 117.4	354.2^AB^ ± 102.5	415.3^A^ ± 136.1
Benthivorous fish*	^**3**^ NPUE (ind. 10 m^−2^)	7.6^A^ ± 2.9	13.0^B^ ± 4.2	13.7^B^ ± 3.9	11.8^B^ ± 3.5	13.5^B^ ± 4.1	14.9^B^ ± 4.3
	^**4**^ WPUE (g 10 m^−2^)	345.2^A^ ± 98.4	666.3^B^ ± 212.9	468.9^A^ ± 149.7	783.2^B^ ± 287.1	814.3^B^ ± 341.9	760.1^B^ ± 209.4
Carnivorous fish*	^**5**^ NPUE (ind. 10 m^−2^)	1.8^AB^ ± 0.4	2.2^A^ ± 0.4	2.5^A^ ± 0.5	1.4^B^ ± 0.3	0.9^C^ ± 0.2	0.4^D^ ± 0.1
	^**6**^ WPUE (g 10 m^−2^)	635.9^AB^ ± 189.2	456.1^A^ ± 105.6	473.1^A^ ± 94.7	653.4^B^ ± 173.8	257.6^C^ ± 74.6	125.1^D^ ± 31.7

*Values in the rows with the same superscripts are not significantly different by the non-parametric Kruskal–Wallis test (ANOVA, *N* = 24, df = 5, *p* < 0.05); ^**1**^*H* = 15.62, *p* = 0.0318; ^**2**^*H* = 16.08, *p* = 0.0283; ^**3**^*H* = 14.57, *p* = 0.0406; ^**4**^*H* = 15.03, *p* = 0.0337; ^**5**^*H* = 20.16, *p* = 0.0063; ^**6**^*H* = 19.47, *p* = 0.0076

The ichthyofauna was represented by twelve fish species (Table S2). The quantitative structure in both ponds was dominated by planktivorous fish. The main representative of benthivores was adult roach, and predators were represented by pikeperch and pike, as well as perch (> 200 g ind.^−1^). In 1-CH, temporal changes in the ichthyofauna were observed, including a decrease in the percentage of planktivorous fish in the range between 82.7 ± 2.5% (2018) and 61.3 ± 3.1% (2020), while an increase in the percentage of benthivorous fish in the range between 14.0 ± 2.1 and 32.7 ± 3.5%, and predatory fish in the range between 3.3 ± 0.2 and 6.0 ± 0.4%. In the 2-CH pond, the percentage of planktivorous and benthivorous fish in the quantitative structure was relatively stable, with a steady decrease in the percentage of predatory fish in the range between 3.1 ± 0.3 (in 2018) and 0.7 ± 0.1% (in 2020). The ichthyobiotic indices of quantity (NPUE) and biomass (WPUE) of all fish (total) in pond 1-CH ranged from 67.5 ind. 10 m^−2^ and 1217.4 g 10 m^−2^ in 2020 to 107.8 ind. 10 m^−2^ and 1530.8 g 10 m^−2^ in 2019 (Table 3). On the other hand, in pond 2-CH, the total fish density index (NPUE) increased in the range from 78.0 ind. 10 m^−2^ (2018) to 103.1 ind. 10 m^−2^ (2020) with a decrease in total fish biomass (WPUE) in the range from 1738.8 g 10 m^−2^ (2018) to 1300.5 g 10 m^−2^ (2020).

**Fig. 2:**
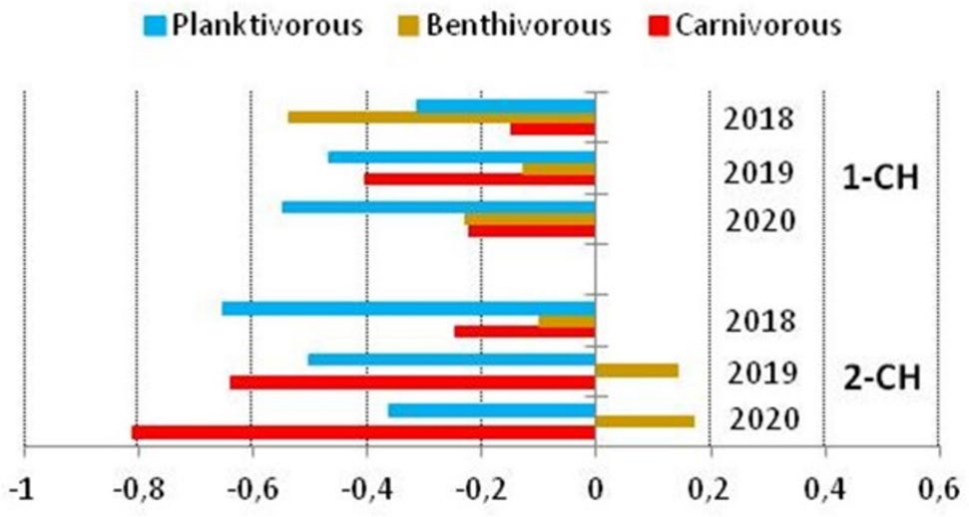
Temporal and spatial changes of the trophic group status (TGS) index in the ichthyofauna of Chabielice pond (1-CH and 2-CH) in 2018–2020

The biomass relationship between fish groups is reflected in the TGS index. In pond 1-CH, a decline in the status of planktivorous fish was visualized in the TGS range from − 0.313 (2018) to − 0.548 (2020). (Fig. 2). In addition, in 2020, the status of benthivorous and carnivorous fish in the TGS range was balanced: − 0.229 and − 0.223. Thus, there was no dominance of biomass of any trophic group in the fish community. In contrast, in pond 2-CH, there was a trend of increase in the status of planktivorous fish at the TGS level from − 0.652 (in 2018) to − 0.361 (in 2020), while the importance of carnivorous fish decreased from − 0.248 (in 2018) to − 0.808 (in 2020). At the same time, in 2019 and 2020, the dominance of benthivorous fish in the total biomass of ichthyofauna was observed with TGS values of: 0.142 and 0.169 (Fig. 2).

In pond 1-CH, the percentage of planktivorous fish decreased in contrast to carnivorous fish and with an increase in the percentage of pikeperch. In pond 2-CH, on the other hand, the trend of change was the opposite, with a decreasing percentage of pikeperch.

This species is characteristic of turbid waters, unlike perch and pike, which are less effective at eliminating their prey in ecosystems with turbid waters (Kokkonen et al. 2019). Such phenomena of direct and indirect interactions have been well understood and described as a result of research using biomanipulation techniques (Gopal et al. 2020). Thus, the observed relationships reflect the effect of ichthyofaunal structure on the zooplankton community by reducing the abundance of planktivorous fishes by predatory fishes (Pal and Chatterjee 2012; 2015), but are not fully consistent with the concept of bottom-up and top-down control of primary production (Makler-Pick et al. 2017; Li et al. 2020). At the same time, long-standing biomanipulation experiments show that moderate levels of planktivorous fish biomass can optimize and stabilize water quality indicators (Wissel et al. 2000). Therefore, it is reasonable to assume that plankton may have been limited by the large amount of suspended solids, including both mechanical and shading aspects. According to Laurenceau-Cornec et al. (2015) and Sobolev et al. (2009), these are mainly the sinking of phytodetrital aggregates and light limitation. Similar conclusions are found in the studies of Napiórkowska-Krzebietke and Skrzypczak (2021). It is also possible that plankton have a bidirectional effect and influence sedimentation rates through the process of biological flocculation. According to research, flocculation may be one of the key factors in the sedimentation process of suspended matter, and biological particles such as phytoplankton, zooplankton, and detritus are important contributors to this process (Maggi 2009; Ming and Gao 2022). The role of zooplankton in the flocculation process is not well understood. The opposite is true for planktonic algae. According to Deng et al. (2019), the size of organic aggregates is determined by the ratio of algal concentration to suspended particle concentration. Salinity alone may not induce flocculation of mineral particles, while diatoms are important for this process (Deng et al. 2019). In the ponds of the Chabielice sedimentation complex in 2018–2020, the zooplankton structure was dominated by Rotifera with a predominance of species from the genera *Ascomorpha* and *Polyarthra*. The phytoplankton was dominated by *Bacillariophyta* (mainly from the genus Ulnaria sp.) and *Ochrophyta* (from the genera *Uroglena* sp. and *Dinobryon* sp.). According to the researches of Napiórkowska-Krzebietke and Skrzypczak (2021) and Skrzypczak and Napiórkowska-Krzebietke (2020) in ponds supplied with water of the same origin (dewatering of the Szczerców opencast mine), the phytoplankton was dominated mainly by diatoms (41%-67% of the total biomass) of the genera *Cyclotella*, *Nitschia*, and *Ulnaria* usually coexisted with these genera of zooplankton. Thus, it should be assumed that diatoms had a potentially positive effect on the efficiency of sedimentation processes in the Chabielice sedimentation complex, but this does not explain the differential nature of sedimentation in individual ponds.

**Fig. 3:**
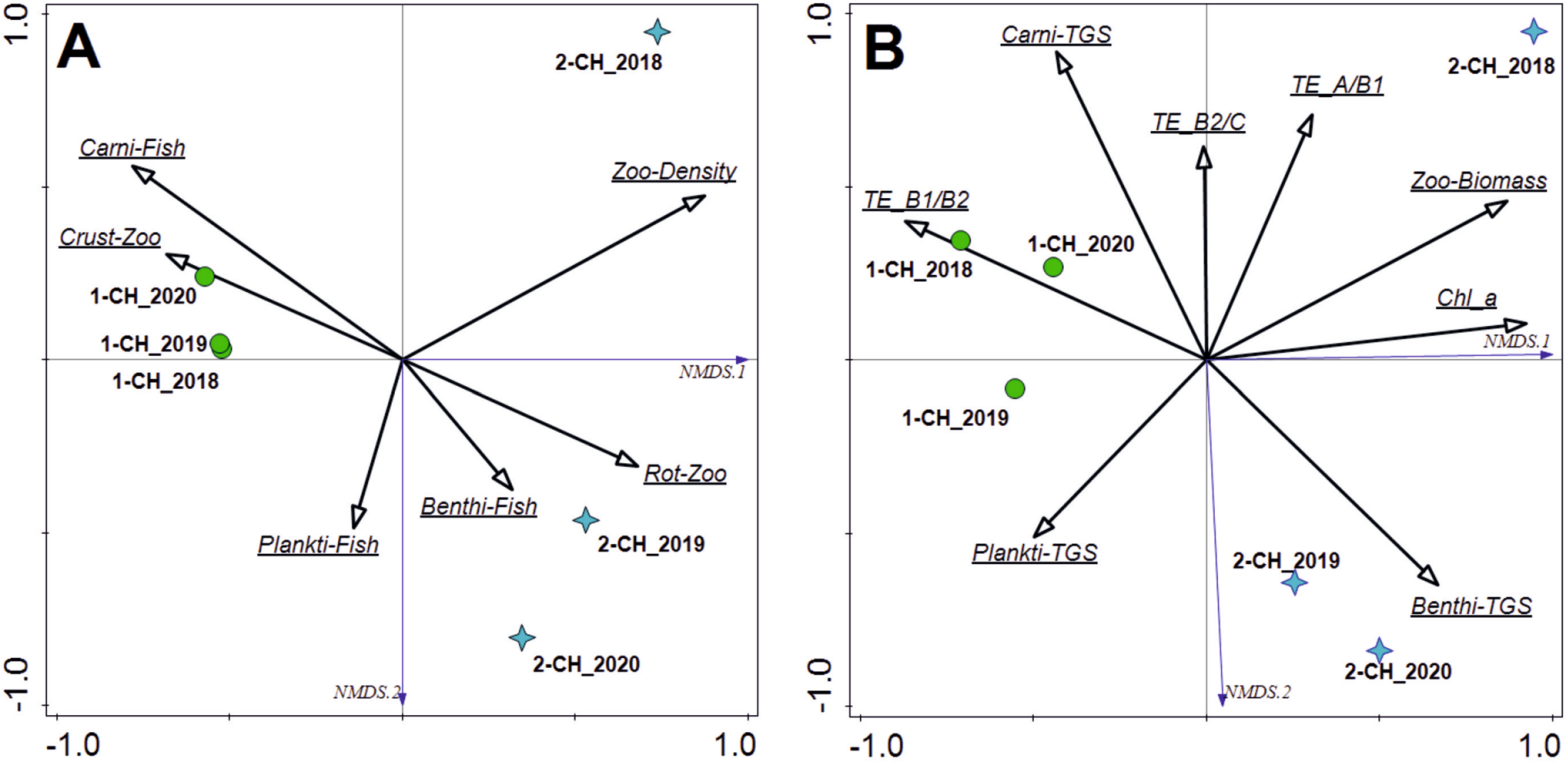
The NMDS triplot based on (**A**) biotic density indices and (**B**) biotic biomass indices with chlorophyll a concentration (Chl_a) and reduction rates of total suspended solids (reduction index of total suspended solids between different sections of the sediment pond: *TE_A/B1*—inflow zone vs. front of the central zone; *TE_B1/B2*—front section vs. rear section of the central zone; *TE_B2/C*—rear section of the central zone vs. plant filter zone) in the ponds of the Chabielice sedimentation complex (1-CH and 2-CH) in the years 2018–2020 (Abbreviations: *Zoo-Density*—total zooplankton density; *Zoo-Biomass*—total zooplankton biomass; *Crust-Zoo*—percentage of crustaceans in zooplankton; *Rot-Zoo*—percentage of rotifers in zooplankton; *Plankti-Fish*—percentage of planktivorous fish in ichthyofauna; *Benthi-Fish*—percentage of benthivorous fish in ichthyofauna; *Carni-Fish*—percentage of carnivorous fish in ichthyofauna; *Plankti-TGS*—status of planktivorous fish; *Benthi-TGS*—status of benthivorous fish; and *Carni-TGS*—status of carnivorous fish)

Biotic indices of structure and density of zooplankton and fish were analyzed in reduced ordination space. NMDS analysis with a stress value of 0.00022924 separated three different groups of samples (Fig. 3A). The first group included all samples collected in the 1-CH pond. For them, the key factor for separation was the steady upward trends in the proportions of crustaceans in the zooplankton and carnivorous fish in the ichthyofauna structure. The second group was formed by samples collected in the 2-CH pond in 2019–2020. They were separated due to the stability of the structure of the percentage of planktivorous and benthivorous fishes in the ichthyofauna and the relatively high and constant percentage of rotifers in the zooplankton. A separate position in the ordination space was characterized by pond 2-CH in 2018. Compared to the other samples, its separation reflected the high density of zooplankton and the high variability of this biotic index. All multivariate data sets, including biotic factors based on biomass indices and chlorophyll concentration, corresponding to total phytoplankton biomass, versus total suspended solid reduction rates in both chambers and three annual cycles, were tested in reduced ordination space with NMDS. The analysis with a stress value of 0.00048929 made it possible to distinguish three separate groups of samples (Fig. 3B). The separation of the samples from pond 1-CH was mainly due to the high value of the TSS reduction index in the central zone of the clarifier (TE_B1/B2). The NMDS 1 axis explained 75.4% of the total variation. Total zooplankton biomass (0.868) and chlorophyll a concentration (0.925) were most positively correlated with this axis. The index of total suspended solid reduction in the central zone showed the strongest negative correlations with NMDS 1 (− 0.869). It was also strongly correlated positively with the TGS index of carnivorous fish (0.709) and negatively with the TGS index of benthivorous fish (− 0.747). These correlations, together with the low TE_B1/B2 values, confirm the discriminability of the samples from pond 2-CH in 2019 and 2020.

Our results indicate that the likely reason for the different nature of the sedimentation process in 2-CH, including the lower efficiency of the TE rate in its central zone, was the resuspension of sediments due to the activity of fish life. There, an increase in the abundance and biomass of planktivorous fish was correlated with a decrease in the abundance and biomass of predatory fish, combined with a decrease in the proportion of pikeperch and an increase in the proportion of perch among the predators. This most likely contributed to a deeper remodeling of the structure of the ichthyofauna in 2-CH. As a result, the fish community was dominated by benthivorous species, mainly roach and bream. In 2-CH, the biomass of benthic fishes was higher throughout the study period, although quantitatively the differences were not so spectacular. This is confirmed by the increasing TGS of benthivorous fish in 2-CH in 2019 and 2020 (0.142 and 0.169, respectively), supported by the increasing proportion of juvenile roach among planktivorous fish.

The effect of bottom-dwelling fish on the resuspension of bottom sediments in shallow waters has been confirmed by Havens (1991), among others. Fish contribute to the turbidity and color of the water and support the cycling of biogenic elements. Thus, they determine the abundance and biomass of phytoplankton (Yahel et al. 2008; Vilizzi and Tarkan 2015; Weber and Brown 2015). This phenomenon is so well understood that it has been modeled (Skulovich et al. 2017). Experimentally, the presence of benthivorous fish has been found to significantly reduce sediment consolidation. Unconsolidated sediments, in turn, are more easily resuspended by wind during wave action (Scheffer et al. 2003). The results of sediment resuspension studies suggest that the remedy is shock therapy by removing fish from the environment. However, the example of sedimentation ponds with a plant filter contradicts such opinions. A similar effectiveness of the plant community in supporting the sedimentation process was found in a study by Moskalski and Sommerfield (2012). Therefore, according to the results of our study, as well as those of Mallo et al. (2010), McCullough et al. (2020), and Skrzypczak and Napiórkowska-Krzebietke (2020), the possibility of extensive aquaculture in sedimentation ponds with a plant filter should not be ruled out without compromising the effectiveness of suspended solid reduction.

## Conclusions

The differences in sedimentation rates are the result of complex mechanisms and the interdependence of biotic factors, including ichthyobiotic factors. Our study showed that the greater influence on sedimentation rates was not so much the abundance of bentophages, but their biomass and the status of this trophic group within the fish community as a whole. Thus, the sedimentation process is favored by stable relationships in the fish community in the absence of dominance by benthivorous fish and a balanced proportion of planktivorous fish. The abundance and biomass of planktivorous fish are controlled by predators that feed effectively in the turbid water body and contribute to the effective functioning of the sedimentation ponds. At the same time, it should be noted that the influence of this fish community on the sedimentation process requires further research. Similarly, further research is needed to clarify the role of the density of small zooplankton, mainly rotifers. A connection between these organisms and the sedimentation process of suspensions in the presence of unconsolidated sediments as a result of fish feeding in the bottom zone cannot be ruled out. Such sediments can be more easily moved by the wind and concentrate around small zooplankton. Thus, having a larger surface area and being affected by the turbulence caused by planktivorous fish feeding, they may remain in the suspended fraction longer. At the same time, it is important to note that the key to the ultimate effectiveness of the reduction process is the filter zone of the plants. The slowing down of the water flow in the macrophyte communities and the lack of physical stimuli for water mixing (lack of wind influence and too shallow water layer for large bentophages) neutralized the potential and actual disturbance of the sedimentation process in the central zone of the sedimentation ponds. This increases the potential and possibilities of using sedimentation ponds for other applications, and the results obtained inspire further observations and research.

## Supplementary information

Below is the link to the electronic supplementary material.

ESM 1 (JPEG 72.0 KB)

ESM 2 (DOCX 57.9 KB)

ESM 3 (DOCX 67.4 KB)

## Data Availability

Data will be made available on request.
