# Supplementary figures and images for: Biotic aspects of suspended solid reduction in sedimentation ponds
Source: Environ Sci Pollut Res Int. 2024 Nov 21;31(56):65066–77. doi: 10.1007/s11356-024-35475-0 (PMC11624212; doi:10.1007/s11356-024-35475-0)

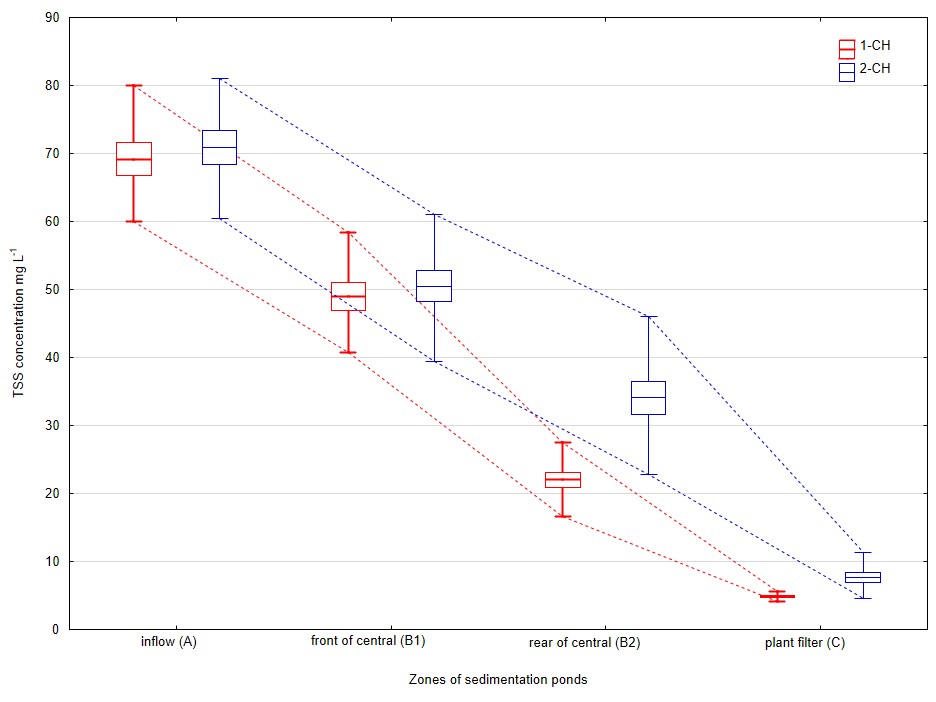

Supplement: Supplementary file 1 — (JPEG 72.0 KB) [file 11356_2024_35475_MOESM1_ESM.jpg]
