# Supplementary material for: Biotic aspects of suspended solid reduction in sedimentation ponds
Source: Environ Sci Pollut Res Int. 2024 Nov 21;31(56):65066–77. doi: 10.1007/s11356-024-35475-0 (PMC11624212; doi:10.1007/s11356-024-35475-0)
Supplement: Supplementary file 2 — (DOCX 57.9 KB) [file 11356_2024_35475_MOESM2_ESM.docx]

**Table S1** Physicochemical parameters of water in the Chabielice sedimentation complex (ponds 1-CH and 2-CH) in the years 2018-2020 (mean of twelve samples, ±SD)

| Parameters | 1-CH | | |  | 2-CH | | |
| --- | --- | --- | --- | --- | --- | --- | --- |
|  | 2018 | 2019 | 2020 |  | 2018 | 2019 | 2020 |
| T (°C) | 18.9  ±2.4 | 17.1  ±4.7 | 17.8  ±3.6 |  | 18.6  ±2.2 | 17.2  ±4.4 | 18.0  ±3.9 |
| DO (mg L^-1^) | 8.8  ±0.6 | 10.2  ±0.7 | 9.4  ±0.6 |  | 9.2  ±0.5 | 10.4  ±0.6 | 9.1  ±0.5 |
| ^1^ SDD (m) | 0.90^AB^  ±0.15 | 0.85^AB^  ±0.15 | 0.95^A^  ±0.10 |  | 0.95^AB^  ±0.15 | 0.80^AB^  ±0.20 | 0.70^B^  ±0.15 |
| ^2^ Turbidity (NTU) | 92.6^A^  ±16.6 | 15.3^B^  ±7.4 | 17.3^B^  ±8.2 |  | 128.6^A^  ±27.4 | 21.4^B^  ±9.8 | 24.8^B^  ±9.3 |
| Reaction (pH) | 7.8  ±0.2 | 8.0  ±0.1 | 7.6  ±0.3 |  | 7.7  ±0.2 | 8.1  ±0.1 | 7.9  ±0.3 |
| TDS (mg L^-1^) | 584.2  ±109.7 | 546.8  ±96.4 | 531.6  ±98.7 |  | 572.3  ±104.6 | 558.1  ±111.3 | 523.8  ±102.9 |
| HCO_3_^-^ (mg L^-1^) | 284.6  ±24.2 | 267.9  ±28.7 | 237.1  ±21.8 |  | 291.1  ±31.2 | 254.7  ±26.3 | 243.2  ±19.4 |
| TOC (mg L^-1^) | 3.5  ±1.4 | 2.9  ±0.9 | 3.7  ±1.2 |  | 3.2  ±1.1 | 3.9  ±0.4 | 4.4  ±0.9 |
| Ca^2+^ (mg L^-1^) | 112.6  ±19.7 | 98.3  ±22.6 | 109.8  ±24.3 |  | 109.4  ±20.2 | 105.6  ±19.7 | 117.7  ±21.6 |
| TP (mg L^-1^) | 0.089  ±0.010 | 0.064  ±0.013 | 0.071  ±0.015 |  | 0.087  ±0.009 | 0.052  ±0.006 | 0.084  ±0.019 |
| TN (mg L^-1^) | 0.401  ±0.119 | 0.395  ±0.231 | 0.376  ±0.204 |  | 0.354  ±0.148 | 0.453  ±0.302 | 0.494  ±0.296 |
| Chl *a* (µg L^-1^) | 3.91  ±1.09 | 3.75  ±3.37 | 4.02  ±2.81 |  | 4.99  ±2.53 | 4.12  ±3.26 | 4.81  ±3.04 |

Values in the rows with the different superscripts are significantly different among the years by non-parametric Kruskal–Wallis test (ANOVA, N = 30. df = 5. p < 0.05); **^1^** *H* = 16.23. *p* = 0.0272; **^2^** *H* = 17.52. *p* = 0.0141
