# Supplementary material for: Biotic aspects of suspended solid reduction in sedimentation ponds
Source: Environ Sci Pollut Res Int. 2024 Nov 21;31(56):65066–77. doi: 10.1007/s11356-024-35475-0 (PMC11624212; doi:10.1007/s11356-024-35475-0)
Supplement: Supplementary file 3 — (DOCX 67.4 KB) [file 11356_2024_35475_MOESM3_ESM.docx]

**Table S2** The structure of zooplankton and fishes (percentage of total density) in the ponds 1-CH and 2-CH of the Chabielice sedimentation complex in the years 2018-2020 (mean of eight samples, ±SD)

| **Parameters** | | **1-CH** | | | **2-CH** | | |
| --- | --- | --- | --- | --- | --- | --- | --- |
|  |  | **2018** | **2019** | **2020** | **2018** | **2019** | **2020** |
| **Zooplankton** | Rotifera | 93.1  ±7.4 | 90.8  ±5.2 | 82.7  ±4.6 | 94.1  ±0.9 | 95.7  ±1.4 | 95.1  ±3.2 |
|  | Crustacea | 6.9  ±0.8 | 9.2  ±1.1 | 17.3  ±2.4 | 5.9  ±1.2 | 4.3  ±0.8 | 4.9  ±1.1 |
| **Planktivorous fish *<10g*** | | **82.7**  ±2.5 | **75.3**  ±2.8 | **61.3**  ±3.1 | **71.0**  ±2.9 | **71.9**  ±3.3 | **74.1**  ±4.1 |
| *Abramis brama* | | 0.4  ±0.1 | 1.0  ±0.2 | 0.9  ±0.2 | - | 0.3  ±0.1 | 0.5  ±0.1 |
| *Blicca bjoerkna* | | 0.3  ±0.1 | 2.2  ±0.3 | 0.2  ±0.1 | - | - | - |
| *Carassius auratus* | | - | 0.2  ±0.1 | - | - | 0.2  ±0.1 | - |
| *Gymnocephalus cernua* | | 2.5  ±0.4 | 5.0  ±1.4 | 7.6  ±1.8 | 20.9  ±1.9 | 12.7  ±1.2 | 13.5  ±1.4 |
| *Leucaspius delineatus* | | - | - | - | 5.5  ±0.8 | 8.5  ±0.9 | 9.6  ±1.4 |
| *Perca fluviatilis* | | 12.6  ±1.4 | 6.8  ±0.9 | 9.9  ±1.5 | 30.5  ±2.7 | 26.6  ±3.3 | 18.7  ±1.6 |
| *Rutilus rutilus* | | 84.2  ±3.9 | 84.8  ±4.7 | 81.4  ±5.4 | 43.1  ±3.3 | 51.7  ±4.2 | 57.7  ±3.9 |
| **Benthivorous fish ≥10g** | | **14.0**  ±2.1 | **21.1**  ±2.8 | **32.7**  ±3.5 | **25.9**  ±2.6 | **26.4**  ±2.1 | **25.2**  ±2.3 |
| *Abramis brama* | | 11.0  ±1.8 | 26.1  ±2.2 | 8.9  ±1.1 | 7.0  ±1.2 | 9.1  ±2.1 | 14.4  ±2.4 |
| *Blicca bjoerkna* | | 1.5  ±0.1 | 10.7  ±1.1 | 2.8  ±0.3 | 4.3  ±0.4 | 6.6  ±0.8 | 7.1  ±0.7 |
| *Carassius auratus* | | 2.2  ±0.2 | 2.1  ±0.3 | 3.8  ±0.6 | 7.0  ±0.9 | 4.1  ±0.4 | 3.0  ±0.2 |
| *Carassius carassius* | | - | - | - | 0.9  ±0.1 | 2.1  ±0.2 | 2.6  ±0.1 |
| *Gymnocephalus cernua* | | 1.5  ±0.3 | 1.7  ±0.1 | 1.6  ±0.3 | 0.6  ±0.2 | - | - |
| *Leuciscus idus* | | - | - | - | 0.9  ±0.1 | 1.2  ±0.2 | 1.5  ±0.2 |
| *Perca fluviatilis (*≤*200g)* | | 9.5  ±2.3 | 1.7  ±0.1 | 17.9  ±2.9 | 3.7  ±0.4 | 0.8  ±0.1 | - |
| *Rutilus rutilus* | | 74.3  ±6.2 | 57.7  ±5.6 | 65.0  ±6.8 | 75.6  ±7.3 | 76.1  ±7.9 | 71.4  ±6.2 |
| **Carnivorous fish** | | **3.3**  ±0.2 | **3.6**  ±0.2 | **6.0**  ±0.4 | **3.1**  ±0.3 | **1.7**  ±0.2 | **0.7**  ±0.1 |
| *Leuciscus aspius* | | - | - | - | 4.0  ±0.2 | 12.5  ±1.2 | - |
| *Esox lucius* | | 6.2  ±0.8 | 12.5  ±0.9 | 8.8  ±1.1 | 8.0  ±0.7 | 18.7  ±1.3 | 14.3  ±1.5 |
| *Perca fluviatilis (>200g*) | | 37.5  ±3.6 | 15.0  ±2.7 | 15.6  ±1.9 | 20.0  ±3.9 | 31.3  ±3.4 | 57.1  ±4.6 |
| *Sander lucioperca* | | 56.3  ±3.9 | 72.5  ±5.6 | 75.6  ±4.6 | 68.0  ±4.0 | 37.5  ±2.6 | 28.6  ±3.7 |
